# COPD Exacerbation: Why It Is Important to Avoid ICU Admission

**DOI:** 10.3390/jcm12103369

**Published:** 2023-05-09

**Authors:** Irene Prediletto, Gilda Giancotti, Stefano Nava

**Affiliations:** 1 Alma Mater Studiorum University of Bologna, Department of Medical and Surgical Science (DIMEC), Via Massarenti 9, 40138 Bologna, Italy; 2 IRCCS Azienda Ospedaliero Universitaria di Bologna, Respiratory and Critical Care Unit, Policlinico S. Orsola-Malpighi di Bologna, Via Albertoni 15, 40138 Bologna, Italy

**Keywords:** COPD exacerbations, acute respiratory failure, intensive care unit, COPD epidemiology, COPD hospitalization

## Abstract

Chronic obstructive pulmonary disease (COPD) is one of the major causes of morbidity and mortality worldwide. Hospitalization due to acute exacerbations of COPD (AECOPD) is a relevant health problem both for its impact on disease outcomes and on health system resources. Severe AECOPD causing acute respiratory failure (ARF) often requires admission to an intensive care unit (ICU) with endotracheal intubation and invasive mechanical ventilation. AECOPD also acts as comorbidity in critically ill patients; this condition is associated with poorer prognoses. The prevalence reported in the literature on ICU admission rates ranges from 2 to 19% for AECOPD requiring hospitalization, with an in-hospital mortality rate of 20–40% and a re-hospitalization rate for a new severe event being 18% of the AECOPD cases admitted to ICUs. The prevalence of AECOPD in ICUs is not properly known due to an underestimation of COPD diagnoses and COPD misclassifications in administrative data. Non-invasive ventilation in acute and chronic respiratory failure may prevent AECOPD, reducing ICU admissions and disease mortality, especially when associated with a life-threating episode of hypercapnic ARF. In this review, we report on up to date evidence from the literature, showing how improving the knowledge and management of AECOPD is still a current research issue and clinical need.

## 1. Introduction

Chronic obstructive pulmonary disease (COPD) is one of the major causes of morbidity and mortality worldwide, with a prevalence of approximately 12% and three million deaths annually [1]. Hospitalization due to acute exacerbation of COPD (AECOPD) is a relevant health problem due to its impact on disease outcomes and health system resources [2]. The one-year mortality after AECOPD requiring hospitalization is 25% [3].

Due to the global impact of the disease, the World Health Organization (WHO) included COPD in the WHO Global Action Plan for the Prevention and Control of Noncommunicable Diseases and the United Nations 2030 Agenda for Sustainable Development [4].

COPD is a common, preventable, and chronic disease characterized by an increase in airflow obstruction and the progressive development of respiratory symptoms, including dyspnea, chronic coughs, increased sputum production, and wheezing. The natural history of COPD accounts for the progressive worsening of respiratory symptoms and flow limitation: the end-stage of the disease is usually represented by respiratory failure. According to the Global Initiative for Chronic Obstructive Lung Diseases (GOLD), AECOPD is defined as an acute worsening of respiratory symptoms needing additional therapy [5].

AECOPD requiring hospitalization represents a negative prognostic factor for mortality [6,7] and is a frequent cause of acute respiratory failure (ARF) [8]. In some cases, ARF due to AECOPD can rapidly worsen, leading to endotracheal intubation and invasive mechanical ventilation. For these reasons, severe AECOPD causing acute or acute-on-chronic respiratory failure often requires admission to an intensive care unit (ICU) [9]. To properly manage patients, one of the most important factors in severe AECOPD is the early evaluation of the degree of the acute respiratory failure. AECOPD is a high-impact comorbidity in critically ill patients and is associated with poorer prognoses [10,11,12].

## 2. Results

### 2.1. AECOPD in ICU Setting: Epidemiology

The prevalence of ICU admission rates ranges from 2 to 19% for all the AECOPD cases requiring hospitalization [13,14,15,16]. Many studies have shown some limitations in collecting these epidemiologic data: the main issues are the heterogeneity of COPD study populations, the different selection criteria for ICU admissions, and the ICU stay durations, which vary according to the healthcare system variabilities among different countries. The median age of ICU admission, according to these studies, is 70–74 years, corresponding to the median age of patients with the more severe stages of the disease [17,18,19]. Data regarding the severity stage of COPD before an ICU admission setting are often missing. This could probably depend on the quite recent standardization of the definition of AECOPD and on the absence of evidence-based treatment guidelines. The prevalence of AECOPD in ICUs is underestimated due to both an underestimation of COPD diagnoses and COPD misclassifications in administrative data [20,21,22]. For inpatients, the diagnosis of COPD is often improperly based on previous history and clinical findings, rather than functional evaluation data (spirometry). In addition, the critically ill conditions of suspected COPD patients admitted to ICUs usually do not allow for performing diagnostic tests. On the other hand, some of the patients classified with AECOPD actually did not have COPD, but another kind of lung disease, such as asthma or interstitial lung disease, so some patients could also be misclassified as having COPD (e.g., acute hearth failure with pulmonary oedema). Following these considerations, the real proportion of AECOPD requiring ICU admission may be underestimated and the real influence of COPD on mortality is potentially unknown.

AECOPD leads to a significant increase in resource utilization and healthcare system costs [23]. Hospitalization for AECOPD is a relevant health problem nowadays due to its impact on disease outcomes [24] and economic and health system resources [16,23]. One of the crucial points in severe AECOPD management is the early recognition of ARF and the correct selection of the treatment approach. Moreover, when AECOPD represents an important comorbidity in critically ill patients, it is associated with an unfavorable prognosis [9,11]. The in-hospital mortality for AECOPD requiring ICU admission is 12–24% [25,26,27]. Approximately 25% and 65% of patients hospitalized for AECOPD die within 1 and 5 years, respectively [3]. A challenge in COPD management is the early admission rate after a recent hospitalization for AECOPD: data form the PROSPERO meta-analysis demonstrated pooled 30-, 60-, 90-, 180-, and 365-day readmission rates of 11%, 17%, 17%, 30%, and 37%, respectively [28].

In previous studies, the principally reported negative prognostic factors for mortality during AECOPD cases admitted to an ICU were: a low mean arterial pressure (MAP); elevated blood urea nitrogen (BUN) [29]; an intubation event [29,30]; a lower value of SpO2 at admission 21; lymphocytes < 0.8 × 109/L; leukopenia; chronic heart failure [30,31]; older age, an initial C-reactive protein (CRP) >7.5 mg/dL; a peak eosinophil to neutrophil ratio (ENR) of >102; and in-hospital complications [32].

A recent published study also depicted APACHE II score, admission albumin levels, admission PaO2/FIO2 ratio, and the use of vasopressor agents during an ICU stay as predictors for mortality [33].

A detailed analysis of the data that emerged from these studies is shown in Table 1.

### 2.2. Long-Term Mortality after ICU-Care in COPD Patients

As previously highlighted, there are no extensive data present in the literature concerning AECOPD prognoses in ICUs. Prognostic data sometimes come from unselected study populations instead of a selected severe AECOPD sub-group. The information we have comes from some studies on long-term mortality after ICU stays. The literature data on long-term mortality are very limited: one study found mortality rates at 6 months, 1 year, and 5 years from hospitalization of 39.0%, 42.7%, and 75.9%, respectively [17,18]. Other data come from studies without a selection of the study population: the 5-year mortality of patients admitted to an ICU due to respiratory illness was 44%, while in the entire ICU population, this ranged from 40 to 58% [34]. For AECOPD in an ICU setting requiring invasive mechanical ventilation, the 1-year mortality rate was 40.5–48.6% [35,36] and the 3-year mortality rate was 63.5% [35], while the 1-year mortality of COPD patients treated with non-invasive ventilation (NIV) in an ICU setting was 49.1% [37]. For COPD patients with a “do not intubate” decision receiving NIV in an ICU, there was a 1-year mortality of 70.3% [38]. COPD itself represents a major risk factor for developing a healthcare-associated infection in an ICU setting [39], enhancing the complexity of the disease’s outcome in patients requiring ICU admission. The prolonged use of mechanical ventilation may also contribute to muscle loss [40].

### 2.3. Risk Factors for AECOPD Requiring ICU

So far, no study has identified the potential risk factors and/or aetiology specific to AECOPD requiring ICU admission, so to understand severe AECOPD, we have to refer to the general risk factors and causative agents for AECOPD, which are well illustrated in the literature.

The most relevant risk factors are a history of previous AECOPD exacerbation (>two in the past year), a rapid decline (<100 mL/year) in forced expiratory volume in the first second (FEV1), older age, the grade of severity of airflow limitation, daily coughing and wheezing, reported increasing dyspnoea, cardiovascular comorbidities, chronic bronchitis phenotype, and the presence of bronchiectasis [41,42,43,44,45]. Interestingly, some studies have reported that gastro-oesophageal reflux may also play a role in triggering AECOPD [46,47,48,49,50]. To be more specific, considering the subgroup of AECOPD requiring hospitalization, some risk factors associated with the development of an acute event have been identified: a FEV1 value of <50% of the predicted value; a sedentary lifestyle; comorbidities such as chronic heart failure, dilatative cardiomyopathy, and diabetes; >three emergency room admissions for AECOPD in the past year; an age of >65 years old; the presence of underestimated/unknown chronic respiratory failure; chronic bronchitis phenotype; and an AECOPD event in the previous month [3,42,43,44,51,52]. The risk factors for developing AECOPD are summarized in Table 2.

COPD exacerbations are usually triggered by bacterial infections, but may also be triggered by viruses, allergens, and common pollutants [53,54]. Infections account for up to 80% of all the causes of AECOPD [55,56]. *Haemophilus influenza*, *Streptococcus pneumoniae*, and *Moraxella catarrhalis* are responsible of up to 70% of all AECOPD cases and 90% of bacterial exacerbations [51,57]. Notably, pneumonia in AECOPD also represents a negative prognostic factor for survival [58]. Viruses are also important etiological agents in AECOPD, in particular rhinoviruses, which can often be complicated by subsequent bacterial pneumonia, and influenza viruses [59,60,61,62].

In total, 20–40% of all AECOPD events are triggered by non-infectious conditions, such as cardiovascular comorbidities (in particular heart failure), pulmonary embolisms, extra-pulmonary infections, and pneumothorax [63]. Moreover, some precipitating factors of AECOPD could also be represented by air pollution and/or cold air exposure, allergen exposure, tobacco smoking, and, not negligibly, a poor adherence with COPD therapies, including oxygen [64,65,66,67].

Interactions between all the possible triggers (the pathogens, indoor and outdoor pollution) and a patient’s characteristics increase the inflammation in the lower airways, leading to tissue damage. The aetiological causes associated with AECOPD are described in Table 3. Specifically, for AECOPD patients with hypercapnic ARF (AHRF) requiring ICU admission, we found some indirect data about the causative triggers of exacerbations in the study of Akbas et al. [33], which identifies acute bronchitis, pneumonia, and heart failure as the major causes of such exacerbations. In this study, no data describing the aetiological infectious agents were reported: the only study objective was to identify the mortality predictors of COPD patients requiring ICU admission. Moreover, the major limitation of this study was its retrospective design. Future interest should target both COPD prevention strategies and better understanding the determinants of patient outcomes, in order to avoid early mortality rates after severe AECOPD requiring an ICU setting.

### 2.4. Selection Criteria to ICU-Admission in Severe AECOPD

The ICU setting for severe AECOPD patients allows for the close monitoring of patients in critical condition. The ICU also comprises non-invasive management, such as the management of patients treated with non-invasive mechanical ventilation, including those with COPD exacerbations with severe respiratory acidosis, allowing for a prompt intubation in the case of a worsening of their respiratory or general conditions (e.g., a loss of hemodynamic stability, adequate oxygenation, or good cooperation; the decline of blood gas exchange; and multi-organ failure) [68]. The selection criteria for admitting severe AECOPD patients to an ICU are principally represented by the presence of critical conditions requiring active physiological support, or by the presence of a not yet critical condition requiring intensive care monitoring [69]. The ICU admission criteria are described in Table 4.

The evaluation of an AECOPD patient should start from the severity of the ARF based on the variables depicted in Table 3 and the pre-existing conditions and quality of life of the patient before an ICU hospitalization. Collecting the patient’s history is fundamental in order to evaluate their real-life performances during daily activities and activity limitations, if present. This can guide physicians to better target a patient’s outcome objective and measure it against the patient and/or caregiver’s expectations.

### 2.5. Prognostic Factors for AECOPD Requiring ICU

In general, for all AECOPD events, one of the most important factors that negatively impacts its all-cause mortality rate (both in the short- and long-term) is the presence of AECOPD in the previous 12 months [70]. Re-hospitalization for a new severe event occurs in 18% of AECOPD patients admitted to an ICU. An early hospital re-admission for AECOPD (within 30 days after discharge) is associated with a higher mortality rate [71,72,73].

Regarding in-hospital mortality, there are a lot of conditions representing negative prognostic factors. Poor survival and a higher rate of mortality during hospitalization are significantly associated with males, a lower body mass index, lower arterial oxygen tension (PaO2), severe hypercapnia, and the duration of the ICU hospitalization [74,75]. Additionally, the disease duration, lower serum levels of albumin, and a lower body mass index are associated with poor outcomes [76]. Fatigue, weight loss, and anorexia are daily limitations for patients with severe COPD. Due to these impairments, patients may develop symptoms of depression and/or anxiety. Depression and/or anxiety merit specific questioning when obtaining the medical history of such patients, because they are not only common in COPD, but are also associated with an increased risk of novel exacerbations and a poorer health status [77,78]. Moreover, a central role in COPD history, disease progression, and AECOPD outcomes in an ICU setting is represented by cardiovascular comorbidities, in particular myocardial infarction, stroke, unstable angina, and transient ischemic attack [79,80,81]. Other important conditions that negatively interfere with AECOPD outcomes are the presence of diabetes mellitus, chronic renal failure, and osteoarthritis [82]. Age, a need of IMV [83], the duration of the hospitalization, and complications during the ICU stay [32] contribute to the deterioration of AECOPD patients in an ICU.

Specifically concerning AECOPD requiring an ICU setting of care, few data are reported in the literature describing prognostic factors. A recent cohort retrospective study by Giri et al. [84] illustrated how elevated blood urea nitrogen (BUN) was associated with an increased risk of in-hospital mortality in critically ill patients with AECOPD: subgroups of AECOPD patients with a BUN of > 22 mg/dL had a mean survival time of 15 days (95% CI: 13.6–16.8), whereas the subgroup with a BUN of < 22 mg/dL had a mean survival time of 27 days (95% CI: 19.9–34.2), with *p* < 0.001 (log-rank test). In an older prospective study by Seneff et al. [85], the variables associated with in-hospital mortality included age, the severity of the respiratory and non-respiratory organ system dysfunction, and the length of the hospital stay before the ICU admission. The major factor contributing to prognosis was the development of organ failure added to ARF (60% of total explanatory power) and 180-day outcomes (54% of explanatory power).

For patients aged 65 years or older, mortality doubles in 1 year from 30% to 59%. Interestingly, the physiological respiratory variables (the respiratory rate, serum pH, PaCO_2_, PaO_2_, and alveolar–arterial difference in the partial pressure of oxygen [PAO_2_-PaO_2_]) indicative of advanced dysfunction were more strongly associated with mortality following a hospital discharge (22% of explanatory power) than hospital death rates (4% of explanatory power); the need for mechanical ventilation upon an ICU admission was not associated with mortality. In another retrospective cohort study on 57 patients admitted to an ICU for ARF due to AECOPD, hospital mortality was positively correlated with age, a previous history of MV, the long-term use of oral corticosteroids, the ICU admission albumin levels, the APACHE (acute physiology and chronic health evaluation) II score, and the duration of the hospitalization [18].

### 2.6. Role of Non-Invasive Ventilation in Preventing AECOPD and ICU Rate Admission

Non-invasive mechanical ventilation (NIV) is currently used as a long-term home therapy (LTH-NIV) for patients with chronic stable hypercapnic COPD: after the first AHRF episode, long-term NIV can be used for patients with COPD following a life-threatening AHRF episode requiring acute NIV, if hypercapnia persists following this episode [86,87,88].

According to the European Respiratory Society Guidelines on LTH-NIV for the management of COPD, the possibility of achieving a small reduction in exacerbation rates and hospitalizations with LTH-NIV is suggested [89]. LTH-NIV also improves dyspnoea and exercise tolerance, thus improving the health-related quality of life (HRQL) in COPD. Patients that seem to benefit more from LTH-NIV are those with frequent exacerbations and hospital admissions, especially after a life-threatening episode of acute chronic respiratory failure.

Recommendations for LTH-NIV are conditional, both for its use in stable COPD patients with chronic hypercapnic respiratory failure (CHRF) and patients with COPD following a life-threatening episode of AHRF requiring acute NIV, if hypercapnia persists following the episode (conditional recommendation, low certainty evidence).

Few studies have explored the role of LTH-NIV in improving patients’ prognoses; the positive data come from an accurately selected population of COPD patients with AHRF. Suraj et al. [90], in a prospective cohort study published in 2018, showed how an LTH-NIV group had a 40% reduction in their mortality compared to a non-LTH-NIV group, in COPD patients with known chronic hypercapnic respiratory failure: there were 30 patients with a 6.6% in-hospital mortality rate versus 90 patients with a 11.1% in-hospital mortality rate, respectively, RR = 0.6. Interestingly their data also showed a reduction in the number of hospital admissions (28.6% vs. 84.7%: *p* < 0.05), ICU admissions (7.1% vs. 56.9%: *p* = 0.01), ventilator requirements (3.6% vs. 30.6%: *p* = 0.003), and AHRF cases (7.1% vs. 48.6%: *p* = 0.000) when comparing the LTH-NIV group to the non-LTH-NIV group. No significant changes in lung function and exercise tolerance were reported. Other important data strongly in favor of LT-NIV come from a multicentric prospective randomized controlled trial conducted in Germany and Austria, where the authors demonstrated that LTH-NIV reduces the mortality in COPD patients with CHRF [91]. In particular, in stable COPD patients with CHRF, adding LT-NIV reduces their 1-year mortality rate in comparison to patients treated without home NIV, with, respectively, a 1-year mortality of 12% (*n* = 102) vs. 33% (*n* = 93) and a hazard ratio of 0.24 (95% CI 0.11–0.49; *p* = 0.0004). This trial was stopped early due to the evidence of a benefit for the prognoses of the LTH-NIV group versus the controls.

A pilot prospective randomized study demonstrated that the continuation of home NIV after an occurrence of AHRF requiring NIV in COPD patients reduced the need for NIV, intubation, or death after new episode of AHRF [92]: in the first year after hospital discharge, the incidence of subsequent AHRF episodes was 38.5% for the subgroup who prolonged NIV at home (*n* = 23) vs. 60.2% (*n* = 24) for the subgroup discharged with nocturnal home continuous positive airway pressure (CPAP), *p* = 0.039. The lack of a control group receiving neither nocturnal home NIV or CPAP could represent a possible conceptual bias, even if the patients affected by chronic respiratory diseases other than COPD or with obstructive sleep apnea syndrome were excluded from the study. When analyzing studies with fewer selected COPD patients, the survival benefits or a reduction in AECOPD rates are not so clear, as was the case for the RESCUE trial: the authors investigated if adding home nocturnal NIV to COPD patients after an episode of AHRF requiring hospitalization prolonged the time before a hospital readmission or death in the following 12 months. One year after hospital discharge, 65% of the LTH-NIV group versus 64% of the non-LTH-NIV group patients were readmitted to the hospital for respiratory causes or had died, and the time until this event was not different (*p* = 0.85) [93].

Freitas et al. showed the benefit of initiating LTH-NIV for COPD patients after hospitalization for AHRF, in terms of a reduction in the numbers of future exacerbations, but no benefits in terms of mortality were noticed. This study also had no control group: the comparison of the AECOPD rates was made with a year before enrolment [94].

One meta-analysis published in 2021 reported that LTH-NIV for post-hospital AECOPD patients with AHRF significantly decreased their exacerbation frequencies (weighted mean difference −1.74, 95% CI: −2.90 to −0.57, *p* = 0.004), but no difference was found for mortality [95]. A recent Cochrane database systematic review showed that only patients with persistent hypercapnia after a COPD exacerbation could have a clear benefit from using LTH-NIV to prolong their admission-free survival [96]. The authors underlined the importance of future research investigating the use of LTH-NIV in stable COPD patients to strengthen the evidence and better target NIV treatments according to different patients’ phenotypes.

Additionally, the use of NIV in an acute setting for AECOPD in patients with AHRF has a role in reducing the risk of endotracheal intubation and mortality rates. The data suggesting the use of NIV in AHRF are more robust than those for LTH-NIV, with clear clinical evidence of a reduction in life-threatening episodes of AHRF in AECOPD [97].

As experts advise [89,97], when analyzing NIV benefits, we should consider that this treatment use has some limitations due to technical factors, the type of interface chosen, humidification, and patient-related factors such as adherence and the presence of comorbidities that could positively or negatively impact the NIV success (e.g., chronic congestive heart failure and obesity hypoventilation syndrome, or obstructive sleep apnea syndrome versus mental or physical disability, or a patient’s compliance and patient/caregivers education, respectively).

## 3. Conclusions

The burden of mortality rates for AECOPD is not negligible. Nowadays, COPD represents a major cause of morbidity and mortality worldwide, although many improvements have been made in the management of COPD, both in pharmacological treatment and the global approach to end-stage COPD patients [98].

New data for the epidemiology of AECOPD, and specifically for AECOPD in an ICU setting, are unfortunately lacking. Infectious agents seem to be the most frequent and important triggers of AECOPD; pneumonia also acts as a negative prognostic factor [37,69].

Considering the disease management, one of the most important contributions to COPD prognoses has been obtained in recent decades, introducing NIV for the treatment of AECOPD causing AHRF, and, less strongly, with LTH-NIV [89,97].

Better phenotyping of COPD patients could also be meaningful in the context of AECOPD treatment in an ICU setting. Performing randomized controlled trials in an ICU is challenging, due to the complexity of the clinical situation and the instability of the patients. Recently, very promising data on acute settings for COPD have seemed to emerge for high-flow nasal cannulas (HFNC) used as non-invasive forms of respiratory support [99]; hopefully, this technique might lead to some benefits in chronic use [100,101]. HFNC may have a role in the treatment of patients with AHRF who do not tolerate NIV, as ERS experts have suggested [99]. We should underline, however, that the recommendation for these patients is always to perform a trial with NIV prior to use of HFNC. Moreover, HFNC can be used during pauses from NIV instead of conventional oxygen therapy. Further research is needed to strengthen the role of HFNC use instead of NIV in preventing endotracheal intubation and ICU admission for AECOPD patients with ARF.

Another technique that has emerged as an alternative to MV is high-flow veno-venous extracorporeal membrane oxygenation (VV-ECMO). In some cases, AECOPD patients undergoing MV do not achieve the appropriate gas exchange due to an increase in dynamic hyperinflation, and, consequently, alveolar hyperinflation, worsening their hypercapnia. In addition, MV could lead to ventilator-induced lung injury (e.g., barotrauma) and hemodynamic unbalance. ECMO may be helpful in overcoming these limits, providing an external full respiratory and cardiac support and being able to both oxygenate patients and improve hypercapnia [102,103,104].

The best timing and management of ECMO is still under debate and its use is preferable in very selective situations due to the high incidence of severe complications. The strongest data are in favor of using VV-ECMO with a low tidal volume and highly positive end-expiratory pressure, with the patient in prone position, which seems to improve mortality and avoid both IMV and endotracheal intubation in severe AECOPD patients with hypercapnic ARF [105].

Over recent decades, there has been an increase in the research interest in low-flow extracorporeal carbon dioxide removal (ECCO2R). ECCO2R was introduced to avoid IMV improving alveolar ventilation, combining NIV and the use of an extracorporeal device to remove CO_2_ from patient blood [106,107].

This technique could represent a bridge between NIV and IMV, when the gas exchanges are refractory to NIV alone in AECOPD patients with AHRF. The role of ECCO2R is still under debate due to a not negligible incidence of complications and a lack of solid data about its efficacy [108,109].

Finally, preventing COPD and AECOPD is an urgent need worldwide. Tobacco smoking, the first known cause of COPD, and air pollution’s roles in developing chronic respiratory diseases are still not marginal [110,111]. Strategies for reducing COPD incidence and the severity of the disease (including preventing AECOPD) may reduce the amount of severe AECOPD patients requiring an ICU admission, meaning a clear benefit for the patient’s health but also representing a strategy for reducing the economic impacts of COPD management and ICU stays [112,113,114,115,116,117,118,119]. Future research is needed to explore all these unmet questions about COPD.

## Figures and Tables

**Table 1:** Negative prognostic factor for AECOPD requiring ICU admission in previous studies.

Study	Study Type	*N*	Mean Age (Years)	M (*n*/%)	In-Hospital Mortality (%)	Outcome	Negative Prognostic Factor Identified	OR (95%CI)	*p*
Linsuwat C et al. 2013 [29]	observational, retrospecrive, monocentric	217	67.3	102/47	12	in-hospital death	lower MAP	0.91 (0.86–0.96)	<0.001
							elevated BUN	1.06 (1.01–1.12)	0.04
							intubation event	6.12 (1.24–30.87)	0.03
Chen PK et al. 2019 [32]	retrospective observational case-control, monocentric	146	84 (IQR 78–87)	126/86.3	16.4	in-hospital death	age	1.12 (1.03–1.23)	0.011
							Initial CRP > 7.5 mg/dL	4.52 (1.27–16.04)	0.02
							Peak ENR × 102 on days 8–14	0.22 (0.08–0.63)	0.005
Cao Y et al. 2021 [30]	observational, retrospecrive, monocentric	384	78.2 ± 8.2 SD	280/72.9	11.5	in-hospital death	requiring IMV	30.31 (8.29–110.74)	<0.001
							chronic heart failure	7.63 (2.27–25.64)	0.001
							White blood cell count <4 × 10^9^/L	5.77 (1.05–31.74)	0.044
							Lymphocyte count <0.8 × 10^9^/L	3.60 (1.10–11.76)	0.034
Sandau C et al. 2022 [31]	observational, retrospecrive, multicentric	289	74.8 (IQR 69.6–81.8)	98/34	18 *	less than 4 Days Alive and Out of Hospital within 14 days from admission	SpO2 < 88% within first 24 h	2.4 (1.2–4.8)	0.02
							male gender	1.8 (1.0–3.1)	0.034
Akbaş T et al. 2023 [33]	observational, retrospecrive, multicentric	100	71.6	59/59	29	ICU mortality	APACHE II score admission PaO_2_/FiO_2_ ratio vasopressor use during ICU stay	1.2 (1.0–1.3) 0.99 (0.98–0.99) 8.3 (1.7–47.2)	0.026 0.046 0.011


						90-days mortality rates	APACHE II score Admission albumin level	1.1 (1.0–1.2)	0.011
								0.17 (0.06–0.5)	0.002

Abbreviations: MAP means arterial pressure; BUN blood urea nitrogen; IMV invasive mechanical ventilation; CRP C-reactive protein; ENR eosinophil-to-neutrophil ratio; APACHE II score = Acute Physiology and Chronic Health Evaluation II score. * Dead after 24 h, but within 30 days. 2 subjects died within 24 h after hospital admission.

**Table 2:** Risk factors for COPD exacerbations (A—considering COPD generally. B—risk factors for AECOPD requiring hospitalization).

Major Risk Factors of COPD Exacerbations	Major Risk Factors of AECOPD Requiring Hospitalization
Previous AECOPD exacerbation (≥2 in the past year)	FEV1 < 50% of predicted value
Rapid FEV1 decline (>100 mL/yr)	Sedentary
Age	Comorbidities
Airflow limitation severity	chronic heart failure
Daily cough and wheezing	dilatative cardiomyopathy
Increasing dyspnea	diabetes mellitus type 2
Cardiovascular comorbidities	>3 ER admission for AECOPD in the past year
Chronic bronchitis phenotype	Age > 65 years old
Bronchiectasis	Underestimated chronic respiratory failure
Gastro-esophageal reflux	Chronic bronchitis phenotype
	AECOPD in the Previous month

Abbreviations: AECOPD—acute exacerbations of chronic obstructive pulmonary disease; FEV1—forced expiratory volume in the first second; and ER—emergency department.

**Table 3:** Schema of major etiological triggers of AECOPD.

Type	Frequency Among All AECOPD	Agent
infectoius exacerbations	60–80%	
	divided in:	
	70–85%	*Heamophilus influentiae*
		*Streptococcus pneumoniae*
		*Moraxella catarrhalis*
	(frequent patogens)	Viruses
		*Influenza/parainfluenza viruses*
		*Respiratory syncytial virus*
		*Rhinoviruses*
		*Coronaviruses*
		*Adenovirus*
		*Picornavirus*
		*Metapneumovirus*
	15–30%	*Pseudomonas aeruginosa*
		*Stenotrophomonas* spp.
		*opportunistic Gram-negatives (Klebsiella pn, E.Coli)*
	(less frequent patogens)	*Staphylococcus aureus*
		*Mycoplasma pneumonias*
		*Clamydia pneumoniae*
non-infectoius exacerbations	20–40%	heart failure
		pulmonary embolism
		extra-pulmonmary infections
		pneumothorax
		air pollutants
		Nitrogen dioxide
		Particulates (PM10)
		Sulphur dioxide
		Ozone
		passive tobacco smoking
		allergen exposure
		tobakko smoking
		non compliance with COPD terapies, including oxygen

**Table 4:** Clinical conditions to consider in AECOPD patients for ICU admission.

Parameters To Consider to Admit in ICU a Patients with AECOPD
Very severe symptoms (dyspnoea) that respond inadequately to initial emergency therapy Acute respiratory failure with use of accessory respiratory muscles and change in mental status Persistent/worsening hypoxemia (paO2 < 40–45 mmHg) Persistent/worsening severe respiratory acidosis (pH < 7.25) Worsening of respiratory acidosis despite non-invasive mechanical ventilation Need for invasive mechanical ventilation Hemodynamic instability (need for vasopressors) High-risk comorbidities or conditions (pneumonia, heart failure, cardiac arrhythmia, chronic renal failure, liver failure, immunodeficiency, or malignancy) Changes in mental status (confusion, lethargy, or coma) Other concomitant organ failure (e.g., acute renal failure) Age > 65 years old Depression Absence of care giver History of AECOPD requiring intubation and mechanical ventilation
